# Identification of potential biomarkers and pathogenesis in neutrophil-predominant severe asthma: A comprehensive bioinformatics analysis

**DOI:** 10.1097/MD.0000000000030661

**Published:** 2022-09-23

**Authors:** Shuanglan Xu, Zi Chen, Linyang Ge, Chenhui Ma, Quan He, Weihua Liu, Liuchao Zhang, Linfu Zhou

**Affiliations:** a Department of Respiratory and Critical Care Medicine, The First Affiliated Hospital, Nanjing Medical University, Nanjing, Jiangsu, China; b Institute of Integrative Medicine, Nanjing Medical University, Nanjing, Jiangsu, China.

**Keywords:** bioinformatics, biomarker, neutrophil, pathogenesis, severe asthma

## Abstract

Airway neutrophilia has been associated with asthma severity and asthma exacerbations. This study attempted to identify biomarkers, pathogenesis, and therapeutic molecular targets for severe asthma in neutrophils using bioinformatics analysis.

**Methods:**

Fifteen healthy controls and 3 patients with neutrophilic severe asthma were screened from the Gene Expression Omnibus (GEO) database. Based on the analysis of differentially expressed genes (DEGs), functional and pathway enrichment analyses, gene set enrichment analysis, protein–protein interaction network construction, and analysis were performed. Moreover, small-molecule drug candidates have also been identified.

**Results:**

Three hundred and three upregulated and 59 downregulated genes were identified. Gene ontology function enrichment analyses were primarily related to inflammatory response, immune response, leukocyte migration, neutrophil chemotaxis, mitogen-activated protein kinase cascade, Jun N-terminal kinase cascade, I-kappaB kinase/nuclear factor-κB, and MyD88-dependent toll-like receptor signaling pathway. Pathway enrichment analyses and gene set enrichment analysis were mainly involved in cytokine-cytokine receptor interaction, the TNF signaling pathway, leukocyte transendothelial migration, and the NOD-like receptor signaling pathway. Furthermore, 1 important module and 10 hub genes (CXCL8, TLR2, CXCL1, ICAM1, CXCR4, FPR2, SELL, PTEN, TREM1, and LEP) were identified in the protein–protein interaction network. Moreover, indoprofen, mimosine, STOCK1N-35874, trapidil, iloprost, aminoglutethimide, ajmaline, levobunolol, ethionamide, cefaclor, dimenhydrinate, and bethanechol are potential drugs for the treatment of neutrophil-predominant severe asthma.

**Conclusion:**

This study identified potential biomarkers, pathogenesis, and therapeutic molecular targets for neutrophil-predominant severe asthma.

## 1. Introduction

Asthma is a chronic inflammatory airway disease with susceptibility involving multiple genes and a complex network of inflammatory cells, inflammatory mediators, and cytokines. It is characterized by airway inflammation, airway hyper-responsiveness, and airway remodeling.^[1]^ Excess morbidity, mortality, and economic costs of asthma remain major global public health problems, causing substantial social and individual burdens worldwide.^[2,3]^ Nowadays, established evidence confirmed the diversity and heterogeneity of asthma among the pathogenesis, symptoms, risk factors, disease severity, response to therapies, and prognosis.^[4,5]^ According to Global Initiative for Asthma (GINA) guideline,^[6]^ severe asthma is defined as asthma that has not been controlled despite high doses of inhaled corticosteroid (ICS) and long-acting β2 agonists (LABA), or those that require high doses of ICS-LABA to maintain control.^[7]^ Although severe asthma constitutes only 5%–10% of asthma cases, it is associated with a greater burden than mild asthma.^[6–8]^ No cure for severe asthma or method to prevent it has been established.

Based on granulocyte patterns in bronchoalveolar lavage fluid (BALF), severe asthma has been divided into 4 phenotypes of inflammatory subtypes: isolated eosinophilia, isolated neutrophilia, mixed granulocytic, and pauci-granulocytic.^[9,10]^ Interestingly, neutrophils, but not eosinophils, were increased in induced sputum samples of severe asthmatics, suggesting increased infiltration of neutrophils into the airway.^[11–13]^ Previous studies confirmed that neutrophil counts and airway inflammation were strongly associated with severe asthma phenotypes when compared with mild-to-moderate asthma.^[14,15]^ Therefore, neutrophils may play an important role in the pathogenesis of severe steroid-resistant asthma.

Glucocorticosteroids are now generally being successfully used as anti-inflammatory agents to treat asthma; however, they have no effect on neutrophilic asthma.^[16,17]^ The mechanism underlying neutrophilia and its involvement in the pathogenesis of severe asthma remain unclear.^[18]^ Thus, there is an urgent need to discover novel biomarkers and molecular mechanisms to provide new targets for the effective prevention and treatment of neutrophil-predominant severe asthma. Bioinformatics analysis, a method of analyzing gene expression, has confirmed to be an efficient approach to identify hub genes and potential biomarkers for disease diagnosis, treatment, and prevention.^[19]^ This study attempted to identify biomarkers, pathogenic factors, and therapeutic molecular targets for neutrophil-predominant severe asthma using bioinformatics analysis, in order to deliver more precise diagnosis and treatment options.

## 2. Materials and Methods

### 2.1. Ethical approval

The participant data from microarray dataset were based on online datasets, thus, no ethical approval and patient consent are required.

### 2.2. Microarray data

Based on the platform of GPL6104 platform (Illumina humanRef-8 v2.0 expression beadchip), the microarray dataset of accession number GSE137268 was obtained from the Gene Expression Omnibus (GEO) datasets (https://www.ncbi.nlm.nih.gov/).

### 2.3. Differential expression analysis

The GEO2R online web tool (http://www.ncbi.nlm.nih.gov/geo/geo2r) based on R package^[20]^ was used for differentially expressed gene (DEGs) analysis, and the significant DEGs were identified based on the thresholds of |log_2_ fold change | value > 1 and adjusted *P* value < 0.05. DEGs with log_2_ FC > 1 were considered upregulated, and those with log_2_ FC < 1 were classified as downregulated. Volcano and heatmaps were generated to show the characteristics of the DEGs.

### 2.4. Function and pathway enrichment analyses

Gene ontology (GO, http://www.geneontology.org/) functional enrichment analysis was categorized into 3 domains: biological process, cellular component, and molecular function. The Kyoto Encyclopedia of Genes and Genomes (KEGG, http://www.kegg.jp/) database contains information about genes, systems, and chemicals.^[21,22]^ GO and KEGG functional and pathway enrichment analyses for the DEGs were performed using the online tool David Bioinformatics Resources 6.8 (https://david.ncifcrf.gov/),^[23]^ and the top 10 categories were identified using R software. Statistical significance was set at *P* < .05.

### 2.5. Gene set enrichment analysis (GSEA)

We analyzed the association between the expression of upregulated and downregulated hub genes using GSEA 4.0. GSEA was conducted to obtain the biological pathway from a database to a gene set.^[24]^ The cutoff criteria were set as nominal *P* < .05 and enrichment score (ES) > 0.4.

### 2.6. Protein–protein interaction (PPI) network construction, module analysis, and hub genes analysis

The Search Tool for the Retrieval of Interacting Genes/Proteins (STRING) database (https://string-db.org/) was used to characterize the protein–protein interaction (PPI) networks of DEGs,^[25]^ and a comprehensive correlation score > 0.15 as a threshold condition. Cytoscape version 3.6.1 (http://www.cytoscape.org/)^[26]^ was used to screen key modules by Molecular Complex Detection (MCODE) app (score ≥ 10), and analysis of the hub nodes named the core gene or core protein by the cytoHubba plugin with the multiscale curvature classification (MCC) algorithm (score ≥ 5).

### 2.7. Identification of small-molecule candidate drugs

The Connectivity Map (CMap) database can reveal the relationship between small-molecule drugs, gene expression levels, and interrelated diseases,^[27]^ which should help scholars to quickly identify genes highly associated with a disease, identify the main chemical structure of a molecule, and summarize the possible directions of the mechanism of drug molecules. The small-molecule candidate drugs may have a therapeutic effect on asthma, according to the DEGs, and the threshold was set according to *P* < .05, and |enrichment|>0.75.

## 3. Results

### 3.1. Characteristics of participants for microarray

The gene expression profile GSE137268 was generated from sputum samples from 15 healthy controls and 54 asthmatics, and the inflammatory phenotypes of asthma were eosinophilic, neutrophilic, pauci-granulocytic, and mixed-granulocytic. In this study, we focused on the difference between 3 neutrophil-predominant severe asthma and 15 healthy controls.

### 3.2. Identification of DEGs

**Figure 1.**
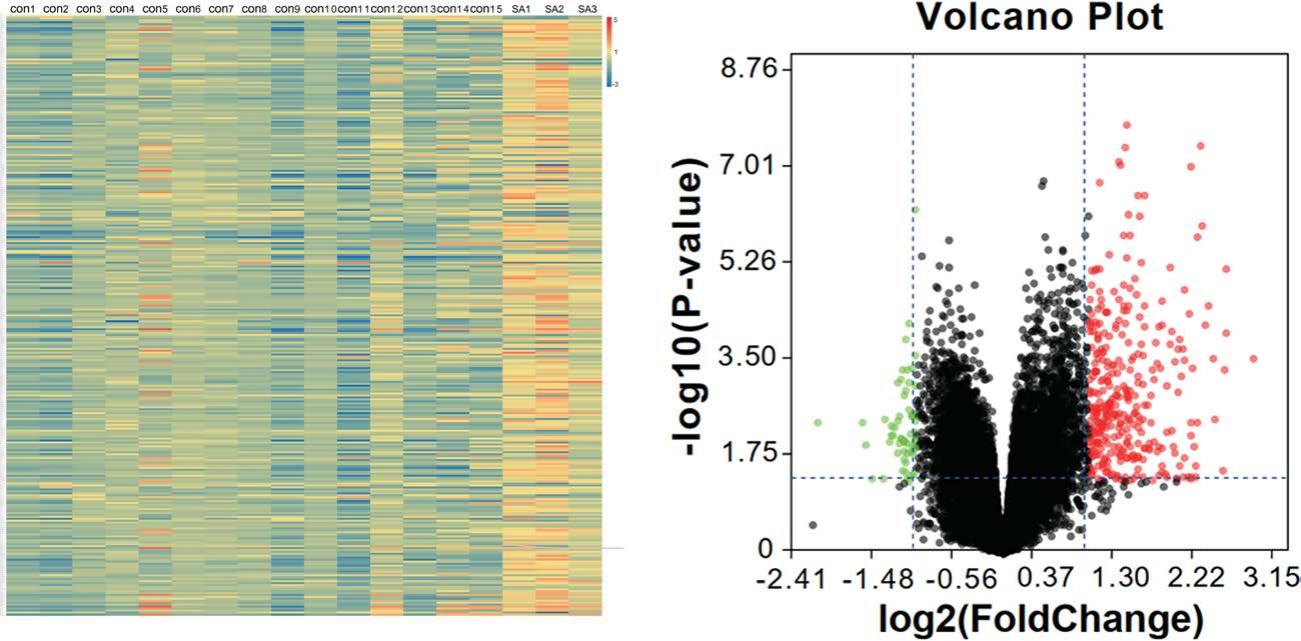
Identification of the significant expression changes of DEGs. (A) The heatmaps of DEGs. (B) Volcano plot of DEGs. Red, blue/green, and black dots represent genes that are upregulated, downregulated and not significantly differentially expressed.

A total of 362 significant DEGs were identified between the healthy controls and neutrophil-predominant severe asthma, which included 303 upregulated and 59 downregulated genes in the latter group (Table 1). Further analysis of these DEGs was performed by creating a heatmap (Fig. 1A) and a volcano plot (Fig. 1B). Complete information is available in Table S1, Supplemental Digital Content 1, http://links.lww.com/MD/H351.

**Table 1:** Identification of DEGs associated with neutrophil severe asthma.

Regulation	Genes
Upregulated (n = 303)	ORM1, PI3, TNFSF14, VNN2, HCAR3, LMOD3, HCAR2, IRAK2, ISG20, SAMSN1, ADORA2A, G0S2, HIST2H2AC, PROK2, FAM200A, NAMPT, ALPP, CAMK2B, ZNF14, TNFAIP6, HSD17B7, TDRD1, RGL4, HIST2H2AA3, CXCR4, FPR2, SOD2, ZNF786, KCNJ2, DNAJC28, DNM1P46, IFITM1, SELL, FAM65B, LPCAT1, HCAR1, TAGAP, CXCR1, ALPL, PHACTR1, LILRA3, FCGR3B, NLRP3, PLAU, FAM63A, TFIP11, H3F3C, MARCKS, CHST15, HIST2H2BE, CXCR2, PDE4B, PELI1, USP49, CMTM2, ANTXR2, FFAR2, IL18RAP, LRG1, CEACAM1, CLEC4E, GABRB1, TMEM71, IL1R2, ADM, ASPRV1, CLC, HLA-C, GBP5, ANKRD22, CLEC4D, WTAP, SIGLEC5, CRISPLD2, TMEM154, MZB1, PADI4, ADGRG3, TREML2, YRDC, CXCL1, HSD17B7P2, CPD, GK, OLIG2, MSX1, LILRA2, C15orf48, PLEK, HES4, SLCO4A1, ZNF223, IFITM2, TNFAIP3, SLC7A5, IDO1, CA4, CDKN2D, KIAA0408, HACD4, CXCL8, CLEC4A, ZNF467, SERPINA1, RASSF5, C15orf39, ATG2A, IER3, MXD1, RASSF2, ORM2, MARCKSL1, LILRA5, DUSP6, GPR84, ERVW-1, C9orf72, IL18R1, SPINK1, PLXNC1, KCNJ15, FPR1, LHX2, LIMK2, LAMB3, KIAA1551, CSF3R, IRAK3, TLR2, ICAM1, S100A9, DYSF, NINJ1, TNFRSF1B, PRKCB, MBOAT7, TMEM158, RAX2, CHST7, CYSTM1, QPCT, SLC16A10, CYFIP2, STX3, TANK, DEFA1B, TNIP1, UPB1, STON1, PTGES, ICAM3, SPAG9, SLC7A11, NDRG1, PIM2, CCR7, SIPA1L1, EDN1, PGLYRP1, BMP6, CDC42EP2, FGF9, RNF150, CEACAM3, CST7, SOCS3, MUCL1, BASP1, MAGEA10, CD93, LCP2, POU5F1, FAM101B, TRIB3, CIDEA, B3GNT8, ADGRE5, CD55, TRIB1, USP10, DCUN1D3, PARVB, ETS2, FAM104B, LEP, NSMAF, TTPAL, PRICKLE1, SNN, L2HGDH, SAPCD2, BMP4, GCA, SAXO1, E2F6, EOMES, GPR132, NBN, FNIP1, STEAP4, SERPINB1, HBP1, GBP1, STOX2, RGS2, OSM, IFITM3, MT1X, RNF19B, ATOH8, TREM1, IRF1, BATF, CDA, TREML4, HIST1H2AC, SCARF1, PDE7B, AQP9, ELF1, SP140, KREMEN1, IL6R, MGAM, ADCY2, TAP1, IGDCC3, SLC15A4, KANK4, LRP10, S100A8, GCH1, PIM3, DLC1, PELI2, ATP6V1B1, CRYAB, CDC42, PTP4A3, IRS2, NOD2, PLK3, HOXA5, ELAVL4, KRT23, SMAP2, ITPRIP, COL8A1, KLHL34, PLEKHO1, RHOH, GCLM, CLDN14, FARSB, PAG1, NKG7, GBP4, ST3GAL4, SEZ6, CACNG2, STX11, OLIG1, NEDD9, IPO11, P2RY8, ST8SIA4, BAZ1A, INPP5A, PRDM8, TNFSF13B, MSRB1, E2F3, HIST1H2BK, CCL3, KIF19, PFKFB3, F5, SEC22B, SMOX, NEDD4L, MLLT6, IRX2, VAMP5, CARD17, GABARAPL2, PHC2, TOM1, IL1RN, BATF2, TSC22D3, LIMS1, NFE2L2, CASP4, CREBRF, CD48, DDIT4, KCNH2, LAMP3, AGMAT, PTEN, CARD19, ZDHHC18, TMEM140
Downregulated (n = 59)	IGFBP2, C8B, ZNF589, GPA33, TMEM74B, SHROOM3, CA2, ECHDC2, SLC47A1, RAPGEF3, MLPH, GPD1, PNPLA7, PROS1, HOXB7, COLEC12, SLC19A3, SH3PXD2A, ACKR3, TAGLN, SPIRE2, GCHFR, ACACB, LGALS3BP, FABP4, FHL1, ENPP3, RDH10, PDE1B, IDUA, GSTT1, PLA2G16, TCEA3, GGA2, TPM2, COL9A2, MARCO, ABCC3, SLC4A11, RMDN3, PKD2L1, GLDN, ST5, EVL, MYB, LY6E, ICOS, C15orf52, FOLR1, PLA2G15, DDIAS, PI4KAP2, DGKQ, FAM156A, SPARC, SLC46A3, PON2, TRPV4, FAM89A

### 3.3. Functional and pathway enrichment analyses of DEGs

GO functional enrichment analysis showed that the main pathways enriched for biological processes were neutrophil functions (including activation, degranulation, chemotaxis, and migration), response to lipopolysaccharide, inflammatory response, immune response, cellular response to interleukin-1 (IL-1), response to interferon-alpha/beta/gamma, leukocyte migration and adhesion, cell apoptosis and proliferation processes, and regulation of signaling pathways, including the inflammatory response, mitogen-activated protein kinase (MAPK) cascade, Jun N-terminal kinase (JNK) cascade, neutrophil chemotaxis, I-kappaB kinase/nuclear factor-κB (NF-κB), myeloid differentiation factor 88 (MyD88)-dependent toll-like receptor (TLR), T-cell receptor, and interleukin-8 (IL-8)-mediated signaling pathways.

**Figure 2.**
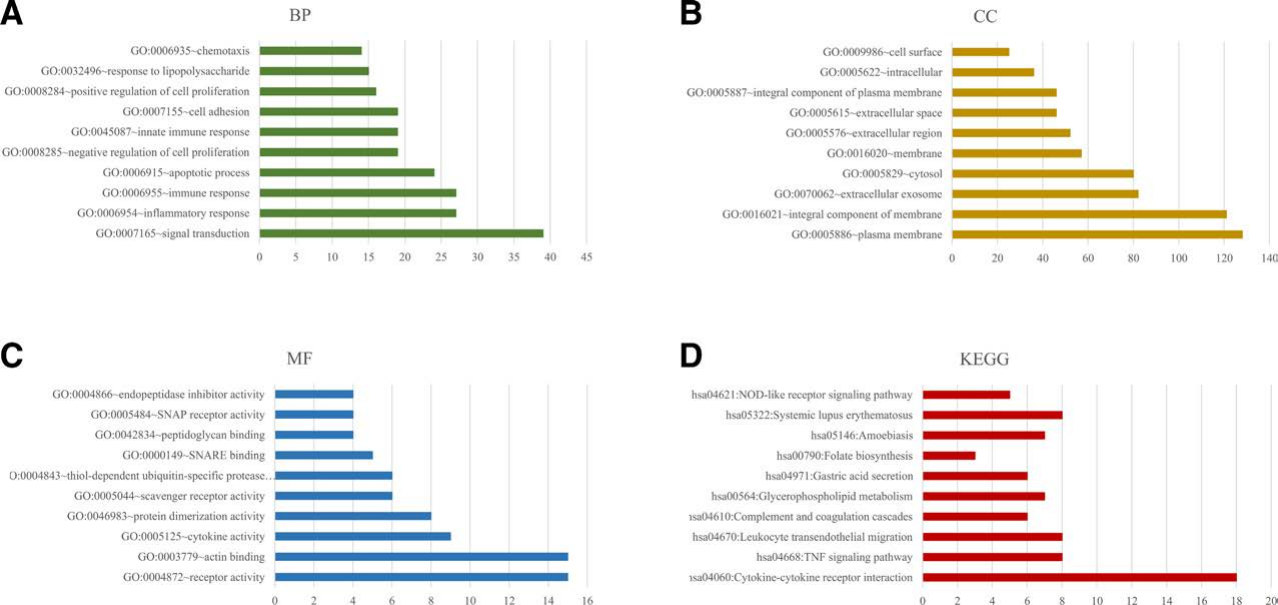
GO functional and KEGG pathway enrichment analysis. Top 10 clusters of (A) BP, (B) CC, (C) MF and (D) KEGG. BP = biological processes, CC = cellular component, MF = molecular function.

Cellular components include intracellular, plasma membrane, extracellular exosome, nucleosome, cortical actin cytoskeleton, and extracellular space. The molecular functions included peptidoglycan binding, IL-8 receptor activity, C-X-C chemokine receptor activity, protein dimerization, and signaling pattern recognition receptor activity. In addition, KEGG pathway analysis revealed enrichment in cytokine–cytokine receptor interaction, nucleotide-binding oligomerization domain (NOD)-like receptor signaling pathway, neutrophil extracellular trap (NET) formation, and viral protein interaction with cytokines and cytokine receptors. Figure 2 shows the top 10 clusters of GO functional enrichment analysis and KEGG pathway enrichment analysis of the DE mRNAs.

#### 3.3.1. GSEA.

**Figure 3.**
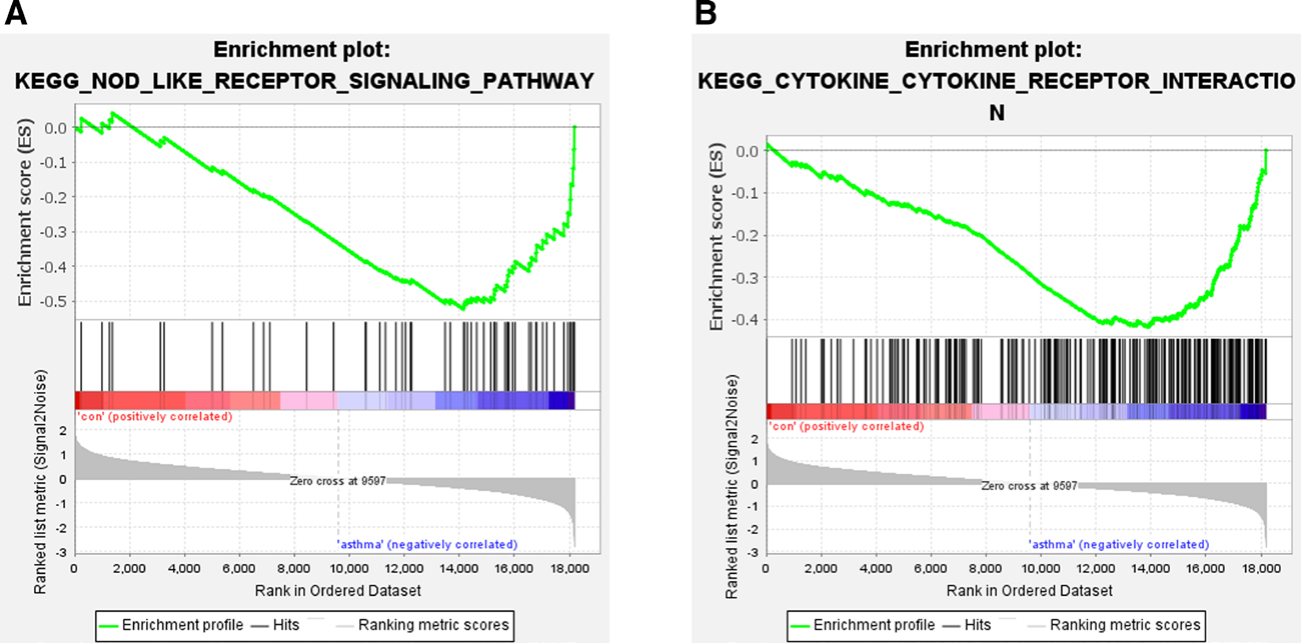
Enrichment plot of GSEA analysis: (A) NOD-like receptor signaling pathways and (B) cytokine–cytokine receptor interaction. GSEA = Gene Set Enrichment Analysis.

GSEA revealed that the upregulated and downregulated hub genes were enriched in the gene sets of “NOD-LIKE RECEPTOR SIGNALING PATHWAY” (Fig. 3A) and “CYTOKINE–CYTOKINE RECEPTOR INTERACTION” (Fig. 3B).

### 3.4. PPI network, module analysis, and hub gene analysis

**Figure 4.**
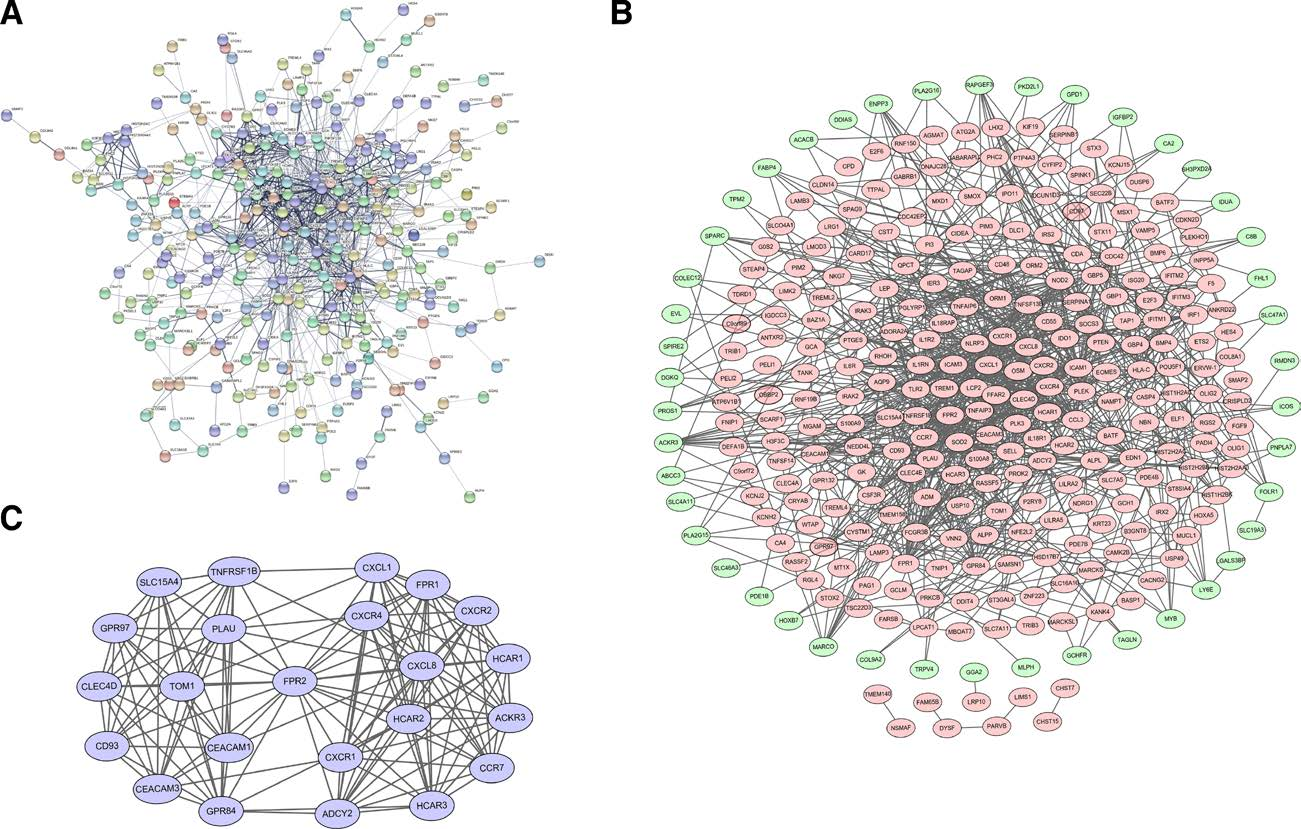
Network of DEGs. (A) PPI network analysis of DEGs, (B) DEGs regulatory network; pink nodes indicate upregulated RNAs, and green nodes indicate downregulated RNAs, and (C) important modules in the PPI network.

The PPI network of the 362 DEGs is presented in Figure 4A, containing 296 nodes and 1073 edges. The upregulated and downregulated genes are shown in Figure 4B. Based on Cytoscape plug-ins, an important module was identified (Fig. 4C), which contained 23 nodes and 144 edges. Additionally, the top 10 hub genes were identified in the PPI network, namely CXCL8, TLR2, CXCL1, ICAM1, CXCR4, FPR2, SELL, PTEN, TREM1, and LEP (Table 2).

**Table 2:** Top 10 Hub genes identified in the PPI network.

Genes symbol	Genes title	Degree	*P* value	Log FC
CXCL8	C-X-C motif chemokine ligand8	56	.0037	1.4647
TLR2	Toll like receptor2	49	.0001	1.3871
CXCL1	C-X-C motif chemokine ligand1	41	.0002	1.5575
ICAM1	Intercellular adhesion molecule1	37	.0004	1.3836
CXCR4	C-X-C motif chemokine receptor4	36	.0002	2.0934
FPR2	Formyl peptide receptor2	36	.0006	2.0709
SELL	SelectinL	29	.0117	1.9684
PTEN	Phosphatase and tensinhomolog	29	.0012	1.0031
TREM1	Triggering receptor expressed on myeloid cells1	28	.0002	1.1544
LEP	Leptin	28	.0204	1.2117

### 3.5. Prediction of small-molecule drugs

Candidate small-molecule drugs were predicted using the CMap database. Negative correlations were considered to indicate drugs with the potential to treat neutrophil-predominant severe asthma, including indoprofen, mimosine, STOCK1N-35874, trapidil, iloprost, aminoglutethimide, ajmaline, levobunolol, ethionamide, cefaclor, dimenhydrinate, and bethanechol (Table 3).

**Table 3:** Results of the connectivity map analysis.

Rank	CMap Name	Mean	N	Enrichment	*P* value	Specificity	Percent
1	Indoprofen	−0.621	4	−0.914	.0001	0	100
2	Mimosine	−0.503	3	−0.877	.00377	0	100
3	STOCK1N-35874	−0.41	2	−0.86	.03929	0.0729	100
4	Trapidil	−0.261	3	−0.834	.00899	0.037	66
5	Iloprost	−0.246	3	−0.824	.01098	0.025	66
6	Aminoglutethimide	−0.309	3	−0.784	.02049	0.0179	66
7	Ajmaline	−0.312	3	−0.782	.02127	0.0709	66
8	Levobunolol	−0.387	4	−0.781	.00475	0.0192	75
9	Ethionamide	−0.378	3	−0.781	.02157	0.0438	66
10	Cefaclor	−0.356	4	−0.778	.00507	0.0119	50
11	Dimenhydrinate	−0.306	4	−0.769	.00573	0.0072	50
12	Bethanechol	−0.233	4	−0.76	.00682	0	50

## 4. Discussion

Asthma is a heterogeneous disease with a consequence of complex gene-environment interactions, and severe asthma is one of the most common causes of death among hospitalized patients.^[8,28]^ However, the biomarkers and therapeutic targets of neutrophil-predominant severe asthma remain poorly understood. In this study, the gene expression profile GSE137268 was generated from the GEO database, in which 362 significant DEGs, including 303 genes that were upregulated in severe asthma and 59 that were downregulated. We then screened candidate important functional and signaling pathways, hub genes, and small-molecule drugs associated with neutrophil-predominant severe asthma via bioinformatic analysis. This analysis provides new targets for the effective prevention and treatment of neutrophil-predominant severe asthma.

Hundreds of statistically significant differences of gene expression were interpreted by the enrichment analyses using GO functions, KEGG pathways, and GSEA. This analytical strategy revealed that the important functions and pathways related to neutrophil-predominant severe asthma include the inflammatory response, neutrophil chemotaxis, and signaling pathways of MAPK cascade, JNK cascade, NF-κB, IL-8-mediated, cytokine–cytokine receptor interaction, MyD88-dependent TLR, NOD-like receptor, and NET formation. In our study, the hub genes in the PPI network included CXCL8, TLR2, CXCL1, ICAM1, CXCR4, FPR2, SELL, PTEN, TREM1, and LEP, which play important roles in the occurrence and development of neutrophil-predominant severe asthma. Some candidate highlights are discussed below.

As summarized in previous studies, the survival of proinflammatory neutrophils is enhanced in children with neutrophil-predominant severe asthma, which is accompanied by increased neutrophil activation and airway release of proinflammatory cytokines and chemokines.^[29]^ The proinflammatory cytokines IL-8 mediate the recruitment and activation of neutrophils and then enhance the migration of neutrophils into airways.^[30]^ Eventually, this leads to increased inflammation of the lungs and severe asthma with airway remodeling.^[31]^

Neutrophils are the major pathogen-fighting immune cells in mammals. As the first line of defense in the innate immune system against infection, neutrophils can protect against a wide range of infectious pathogens.^[32]^ In 2004, the existence of NETs, which are reticular structures released by neutrophil activation, has been confirmed for the first time.^[33]^ High neutrophil counts are found in patients with severe asthma, and NETs are detectable in the BALF and sputum. Interestingly, higher levels of NETs positively correlated with asthma severity and IL-17 levels.^[34–36]^ IL-17A levels are increased not only in severe asthma, but also in other inflammatory diseases with neutrophil recruitment.^[37,38]^ In our previous review, we proposed that NETs are potential therapeutic targets for severe asthma.^[39]^ However, the mechanism underlying the involvement of NETs in neutrophil-predominant severe asthma remains to be explored.

Mitogen-activated protein kinases (MAPKs) are positively involved in the pathobiology of asthma associated with inflammation and remodeling in the airways by activating immune/inflammatory cells and structure-resident cells.^[40,41]^ MAPKs represent a large family of signaling enzymes that include 3 major subgroups: p38, extracellular regulating kinase, and JNK.^[42]^ In 1 study of BALB/c mice exposed to egg ovalbumin to observe neutrophil recruitment to the airway, this pathological process was reversed by p38 MAPK inhibitors.^[43]^ Notably, in vitro experiments also showed that p38 MAPK inhibitors not only synergistically enhance the efficacy of corticosteroids in alveolar macrophages from asthmatic patients but also have a greater effect in patients with corticosteroid-insensitive asthma.^[44,45]^

The transcription factor NF-κB plays an important role in inflammatory and immune responses, given its ability to induce the expression of many inflammatory mediators and their activation by inflammatory stimuli.^[46,47]^ NF-κB was also found to be persistently activated in severe uncontrolled asthma, probably owing to an inflammatory microenvironment in vivo.^[48]^ Conversely, when peripheral blood mononuclear cells (PBMC) were removed from their in vivo environment to in vitro following the addition of a specific NF-κB inhibitor, the production of the proinflammatory cytokine IL-8 was significantly reduced.^[48]^ Therefore, inhibition of the NF-κB pathway is a potential therapeutic target in neutrophil-predominant severe asthma.

Pattern recognition receptors that recognize microbial-associated molecular patterns (MAMPs) trigger an early immune response to pathogens. One class of pattern recognition receptors is the TLR family, which acts as a first line of defense against invading microbes and pathogens in innate and adaptive immune responses. Dysregulation of TLR has also been shown to lead to numerous disease states.^[49]^ Myeloid differentiation factor 88 (MyD88) is a key adaptor protein for TLR, and MyD88 deficiency (MyD88^−/−^) disrupts TLR signaling pathways. Compared with the level in WT controls, IL-17 concentration was significantly reduced in MyD88^−/−^ mice by ovalbumin-induced allergic asthma, and airway neutrophilia in BALF was significantly reduced by α-GalCer instillation. Taken together, these findings suggest that the MyD88-dependent TLR signaling pathway is critical for neutrophil recruitment and IL-17A production in allergic asthma.^[50]^ Another class of molecules that play an important role in the broader control of adaptive immunity and various disease states is the NOD-like receptor (NLR).^[51,52]^ It is confirmed that NLR could as a novel biomarker with adversely links innate and adaptive immunity and leads to allergic disease and asthmatic lung inflammation.^[53]^ Furthermore, airway tolerance is sufficiently blocked by a NOD2 pattern recognition receptor, leading to Th2-driven lung inflammation.^[53]^ Thus, the NLR could be a novel factor conferring susceptibility to the development of allergic asthma. However, the role of NLR in severe asthma remains unclear.

The results for predicting small-molecule drugs with potential efficacy for treating neutrophilic severe asthma identified indoprofen, mimosine, STOCK1N-35874, trapidil, iloprost, aminoglutethimide, ajmaline, levobunolol, ethionamide, cefaclor, dimenhydrinate, and bethanechol. In previous studies, these small-molecule drugs have rarely been studied in neutrophil-predominant severe asthma. Further research is needed to confirm whether these drugs are effective against this disease.

This study had some limitations. First, the results are only based on bioinformatic predictions, and experimental validation in vitro and in vivo is lacking. Moreover, the sample size of the study was relatively small. Thus, the findings of this study should be validated in a larger cohort. Further research is warranted to determine potential biomarkers, pathogenic factors, and therapeutic molecular targets in the generation and development of neutrophil-predominant severe asthma.

## 5. Conclusions

In conclusion, this study identified 3 key genes as potential biomarkers, pathogenic factors, and therapeutic molecular targets for neutrophil-predominant severe asthma. These findings deepen our understanding of the molecular mechanisms underlying the pathogenesis, diagnosis, treatment, and prognosis of neutrophil-predominant severe asthma. For more conformation further extended studies would be required.

## Acknowledgment

The authors would like to acknowledge the GEO database for providing data.

## Author contributions

**Conceptualization:** Shuanglan Xu.

**Formal analysis:** Liuchao Zhang, Quan He, Weihua Liu.

**Software:** Liuchao Zhang, Quan He, Weihua Liu.

**Writing – original draft:** Chenhui Ma, Linyang Ge, Shuanglan Xu.

**Writing – review & editing:** Linfu Zhou, Zi Chen.

## Correction

The funding sentence “This work was supported by grants from the National Key Research and Development Program of China (Grant No. 2018YFC1313600)”, has been replaced by “This work was supported by grants from the National Key Research and Development Program of China (Grant Nos. 2022YFF0710800 and 2018YFC1313600).”

The oricid number, 0000-0003-0691-1676, has been added for Linfu Zhou.

## Supplementary Material


