# Supplementary material for: Identification of potential biomarkers and pathogenesis in neutrophil-predominant severe asthma: A comprehensive bioinformatics analysis
Source: Medicine (Baltimore). 2022 Sep 23;101(38):e30661. doi: 10.1097/MD.0000000000030661 (PMC9509178; doi:10.1097/MD.0000000000030661)
Supplement: Supplementary file 1 [file medi-101-e30661-s001.pdf]

Supplementary Table 1. All of differentially expressed genes.

Gene symbol	Gene name	Regulation	Log FC	P-value
ORM1	orosomucoid 1	up	3.15	0.00
PI3	peptidase inhibitor 3	up	2.90	0.00
TNFSF14	tumor necrosis factor superfamily member 14	up	2.59	0.00
VNN2	vanin 2	up	2.58	0.00
HCAR3	hydroxycarboxylic acid receptor 3	up	2.57	0.00
LMOD3	leiomodin 3	up	2.55	0.03
HCAR2	hydroxycarboxylic acid receptor 2	up	2.44	0.00
IRAK2	interleukin 1 receptor associated kinase 2	up	2.38	0.00
ISG20	interferon stimulated exonuclease gene 20	up	2.34	0.00
SAMSN1	SAM domain, SH3 domain and nuclear localization signals 1	up	2.32	0.00
ADORA2A	adenosine A2a receptor	up	2.29	0.00
G0S2	G0/G1 switch 2	up	2.25	0.00
HIST2H2AC	histone cluster 2, H2ac	up	2.24	0.00
PROK2	prokineticin 2	up	2.23	0.03
FAM200A	family with sequence similarity 200 member A	up	2.22	0.04
NAMPT	nicotinamide phosphoribosyltransferase	up	2.20	0.00
ALPP	alkaline phosphatase, placental	up	2.18	0.04
CAMK2B	calcium/calmodulin dependent protein kinase II beta	up	2.18	0.01
ZNF14	zinc finger protein 14	up	2.17	0.04
TNFAIP6	TNF alpha induced protein 6	up	2.16	0.00
HSD17B7	hydroxysteroid 17-beta dehydrogenase 7	up	2.14	0.04
TDRD1	tudor domain containing 1	up	2.10	0.02
RGL4	ral guanine nucleotide dissociation stimulator like 4	up	2.10	0.00
HIST2H2AA3	histone cluster 2, H2aa3	up	2.09	0.00
CXCR4	C-X-C motif chemokine receptor 4	up	2.09	0.00
FPR2	formyl peptide receptor 2	up	2.07	0.00
SOD2	superoxide dismutase 2, mitochondrial	up	2.05	0.00
ZNF786	zinc finger protein 786	up	2.05	0.03
KCNJ2	potassium voltage-gated channel subfamily J member 2	up	2.03	0.00
DNAJC28	DnaJ heat shock protein family (Hsp40) member C28	up	2.01	0.05
DNM1P46	dynamin 1 pseudogene 46	up	2.00	0.04
IFITM1	interferon induced transmembrane protein 1	up	1.99	0.01
SELL	selectin L	up	1.97	0.01
FAM65B	family with sequence similarity 65 member B	up	1.96	0.01
LPCAT1	lysophosphatidylcholine acyltransferase 1	up	1.95	0.00
HCAR1	hydroxycarboxylic acid receptor 1	up	1.93	0.00
TAGAP	T-cell activation RhoGTPase activating protein	up	1.90	0.00
CXCR1	C-X-C motif chemokine receptor 1	up	1.90	0.04
ALPL	alkaline phosphatase, liver/bone/kidney	up	1.89	0.01
PHACTR1	phosphatase and actin regulator 1	up	1.84	0.00
LILRA3	leukocyte immunoglobulin like receptor A3	up	1.84	0.03
FCGR3B	Fc fragment of IgG receptor IIIb	up	1.84	0.03
NLRP3	NLR family pyrin domain containing 3	up	1.84	0.00
PLAU	plasminogen activator, urokinase	up	1.83	0.00
FAM63A	family with sequence similarity 63 member A	up	1.81	0.04

TFIP11	tuftelin interacting protein 11	up	1.81	0.01
H3F3C	H3 histone, family 3C	up	1.80	0.00
MARCKS	myristoylated alanine rich protein kinase C substrate	up	1.79	0.00
CHST15	carbohydrate (N-acetylgalactosamine 4-sulfate 6-O) sulfotransferase 15	up	1.79	0.01
HIST2H2BE	histone cluster 2, H2be	up	1.76	0.00
CXCR2	C-X-C motif chemokine receptor 2	up	1.76	0.01
PDE4B	phosphodiesterase 4B	up	1.75	0.00
PELI1	pellino E3 ubiquitin protein ligase 1	up	1.73	0.00
USP49	ubiquitin specific peptidase 49	up	1.72	0.05
CMTM2	CKLF like MARVEL transmembrane domain containing 2	up	1.71	0.00
ANTXR2	anthrax toxin receptor 2	up	1.71	0.00
FFAR2	free fatty acid receptor 2	up	1.70	0.01
IL18RAP	interleukin 18 receptor accessory protein	up	1.70	0.01
LRG1	leucine rich alpha-2-glycoprotein 1	up	1.70	0.01
CEACAM1	carcinoembryonic antigen related cell adhesion molecule 1	up	1.67	0.00
CLEC4E	C-type lectin domain family 4 member E	up	1.65	0.00
GABRB1	gamma-aminobutyric acid type A receptor beta1 subunit	up	1.63	0.01
TMEM71	transmembrane protein 71	up	1.63	0.01
IL1R2	interleukin 1 receptor type 2	up	1.63	0.00
ADM	adrenomedullin	up	1.62	0.02
ASPRV1	aspartic peptidase, retroviral-like 1	up	1.62	0.00
CLC	Charcot-Leyden crystal galectin	up	1.62	0.00
HLA-C	major histocompatibility complex, class I, C	up	1.62	0.04
GBP5	guanylate binding protein 5	up	1.61	0.00
ANKRD22	ankyrin repeat domain 22	up	1.61	0.01
CLEC4D	C-type lectin domain family 4 member D	up	1.59	0.00
WTAP	Wilms tumor 1 associated protein	up	1.59	0.00
SIGLEC5	sialic acid binding Ig like lectin 5	up	1.58	0.00
CRISPLD2	cysteine rich secretory protein LCCL domain containing 2	up	1.57	0.01
TMEM154	transmembrane protein 154	up	1.57	0.00
MZB1	marginal zone B and B1 cell specific protein	up	1.57	0.02
PADI4	peptidyl arginine deiminase 4	up	1.57	0.02
ADGRG3	adhesion G protein-coupled receptor G3	up	1.57	0.00
TREML2	triggering receptor expressed on myeloid cells like 2	up	1.56	0.00
YRDC	yrdC N6-threonylcarbamoyltransferase domain containing	up	1.56	0.03
CXCL1	C-X-C motif chemokine ligand 1	up	1.56	0.00
HSD17B7P2	hydroxysteroid 17-beta dehydrogenase 7 pseudogene 2	up	1.56	0.04
CPD	carboxypeptidase D	up	1.55	0.00
GK	glycerol kinase	up	1.54	0.00
OLIG2	oligodendrocyte lineage transcription factor 2	up	1.53	0.00
MSX1	msh homeobox 1	up	1.53	0.02
LILRA2	leukocyte immunoglobulin like receptor A2	up	1.53	0.00
C15orf48	chromosome 15 open reading frame 48	up	1.53	0.00
PLEK	pleckstrin	up	1.52	0.00
HES4	hes family bHLH transcription factor 4	up	1.51	0.02
SLCO4A1	solute carrier organic anion transporter family member 4A1	up	1.51	0.02
ZNF223	zinc finger protein 223	up	1.51	0.04
IFITM2	interferon induced transmembrane protein 2	up	1.50	0.00

TNFAIP3	TNF alpha induced protein 3	up	1.49	0.00
SLC7A5	solute carrier family 7 member 5	up	1.48	0.02
IDO1	indoleamine 2,3-dioxygenase 1	up	1.48	0.00
CA4	carbonic anhydrase 4	up	1.48	0.00
CDKN2D	cyclin dependent kinase inhibitor 2D	up	1.47	0.00
KIAA0408	KIAA0408	up	1.47	0.01
HACD4	3-hydroxyacyl-CoA dehydratase 4	up	1.47	0.05
CXCL8	C-X-C motif chemokine ligand 8	up	1.46	0.00
CLEC4A	C-type lectin domain family 4 member A	up	1.46	0.00
ZNF467	zinc finger protein 467	up	1.45	0.01
SERPINA1	serpin family A member 1	up	1.45	0.00
RASSF5	Ras association domain family member 5	up	1.45	0.00
C15orf39	chromosome 15 open reading frame 39	up	1.45	0.00
ATG2A	autophagy related 2A	up	1.45	0.00
IER3	immediate early response 3	up	1.44	0.00
MXD1	MAX dimerization protein 1	up	1.44	0.02
RASSF2	Ras association domain family member 2	up	1.44	0.02
ORM2	orosomucoid 2	up	1.44	0.00
MARCKSL1	MARCKS like 1	up	1.44	0.01
LILRA5	leukocyte immunoglobulin like receptor A5	up	1.44	0.00
DUSP6	dual specificity phosphatase 6	up	1.44	0.00
GPR84	G protein-coupled receptor 84	up	1.43	0.01
ERVW-1	endogenous retrovirus group W member 1	up	1.43	0.01
C9orf72	chromosome 9 open reading frame 72	up	1.43	0.00
IL18R1	interleukin 18 receptor 1	up	1.42	0.00
SPINK1	serine peptidase inhibitor, Kazal type 1	up	1.42	0.04
PLXNC1	plexin C1	up	1.42	0.00
KCNJ15	potassium voltage-gated channel subfamily J member 15	up	1.41	0.00
FPR1	formyl peptide receptor 1	up	1.40	0.01
LHX2	LIM homeobox 2	up	1.40	0.00
LIMK2	LIM domain kinase 2	up	1.39	0.00
LAMB3	laminin subunit beta 3	up	1.39	0.00
KIAA1551	KIAA1551	up	1.39	0.00
CSF3R	colony stimulating factor 3 receptor	up	1.39	0.05
IRAK3	interleukin 1 receptor associated kinase 3	up	1.39	0.00
TLR2	toll like receptor 2	up	1.39	0.00
ICAM1	intercellular adhesion molecule 1	up	1.38	0.00
S100A9	S100 calcium binding protein A9	up	1.38	0.05
DYSF	dysferlin	up	1.38	0.01
NINJ1	ninjurin 1	up	1.37	0.01
TNFRSF1B	TNF receptor superfamily member 1B	up	1.37	0.00
PRKCB	protein kinase C beta	up	1.36	0.02
MBOAT7	membrane bound O-acyltransferase domain containing 7	up	1.36	0.02
TMEM158	transmembrane protein 158 (gene/pseudogene)	up	1.36	0.00
RAX2	retina and anterior neural fold homeobox 2	up	1.36	0.02
CHST7	carbohydrate sulfotransferase 7	up	1.35	0.01
CYSTM1	cysteine rich transmembrane module containing 1	up	1.35	0.00
QPCT	glutaminyl-peptide cyclotransferase	up	1.35	0.01
SLC16A10	solute carrier family 16 member 10	up	1.34	0.01

CYFIP2	cytoplasmic FMR1 interacting protein 2	up	1.34	0.00
STX3	syntaxin 3	up	1.33	0.00
TANK	TRAF family member associated NFKB activator	up	1.33	0.00
DEFA1B	defensin alpha 1B	up	1.33	0.04
TNIP1	TNFAIP3 interacting protein 1	up	1.33	0.00
UPB1	beta-ureidopropionase 1	up	1.33	0.00
STON1	stonin 1	up	1.33	0.01
PTGES	prostaglandin E synthase	up	1.32	0.00
ICAM3	intercellular adhesion molecule 3	up	1.32	0.04
SPAG9	sperm associated antigen 9	up	1.32	0.00
SLC7A11	solute carrier family 7 member 11	up	1.31	0.00
NDRG1	N-myc downstream regulated 1	up	1.31	0.00
PIM2	Pim-2 proto-oncogene, serine/threonine kinase	up	1.31	0.00
CCR7	C-C motif chemokine receptor 7	up	1.31	0.04
SIPA1L1	signal induced proliferation associated 1 like 1	up	1.30	0.00
EDN1	endothelin 1	up	1.30	0.00
PGLYRP1	peptidoglycan recognition protein 1	up	1.29	0.00
BMP6	bone morphogenetic protein 6	up	1.29	0.00
CDC42EP2	CDC42 effector protein 2	up	1.29	0.00
FGF9	fibroblast growth factor 9	up	1.29	0.01
RNF150	ring finger protein 150	up	1.29	0.01
CEACAM3	carcinoembryonic antigen related cell adhesion molecule 3	up	1.28	0.04
CST7	cystatin F	up	1.28	0.00
SOCS3	suppressor of cytokine signaling 3	up	1.27	0.00
MUCL1	mucin like 1	up	1.27	0.00
BASP1	brain abundant membrane attached signal protein 1	up	1.25	0.00
MAGEA10	MAGE family member A10	up	1.25	0.00
CD93	CD93 molecule	up	1.25	0.04
LCP2	lymphocyte cytosolic protein 2	up	1.25	0.00
POU5F1	POU class 5 homeobox 1	up	1.25	0.02
FAM101B	family with sequence similarity 101 member B	up	1.24	0.00
TRIB3	tribbles pseudokinase 3	up	1.24	0.00
CIDEA	cell death-inducing DFFA-like effector a	up	1.23	0.00
B3GNT8	UDP-GlcNAc:betaGal beta-1,3-N-acetylglucosaminyltransferase 8	up	1.23	0.00
ADGRE5	adhesion G protein-coupled receptor E5	up	1.23	0.00
CD55	CD55 molecule (Cromer blood group)	up	1.23	0.00
TRIB1	tribbles pseudokinase 1	up	1.22	0.00
USP10	ubiquitin specific peptidase 10	up	1.22	0.00
DCUN1D3	defective in cullin neddylation 1 domain containing 3	up	1.22	0.00
PARVB	parvin beta	up	1.22	0.03
ETS2	ETS proto-oncogene 2, transcription factor	up	1.22	0.00
FAM104B	family with sequence similarity 104 member B	up	1.22	0.00
LEP	leptin	up	1.21	0.02
NSMAF	neutral sphingomyelinase activation associated factor	up	1.21	0.00
TTPAL	alpha tocopherol transfer protein like	up	1.21	0.00
PRICKLE1	prickle planar cell polarity protein 1	up	1.21	0.00
SNN	stannin	up	1.21	0.00
L2HGDH	L-2-hydroxyglutarate dehydrogenase	up	1.21	0.03

SAPCD2	suppressor APC domain containing 2	up	1.21	0.01
BMP4	bone morphogenetic protein 4	up	1.20	0.01
GCA	grancalcin	up	1.20	0.00
SAXO1	stabilizer of axonemal microtubules 1	up	1.20	0.00
E2F6	E2F transcription factor 6	up	1.20	0.02
EOMES	eomesodermin	up	1.20	0.01
GPR132	G protein-coupled receptor 132	up	1.19	0.00
NBN	nibrin	up	1.19	0.00
FNIP1	folliculin interacting protein 1	up	1.19	0.00
STEAP4	STEAP4 metalloreductase	up	1.19	0.00
SERPINB1	serpin family B member 1	up	1.19	0.00
HBP1	HMG-box transcription factor 1	up	1.18	0.00
GBP1	guanylate binding protein 1	up	1.17	0.00
STOX2	storkhead box 2	up	1.17	0.01
RGS2	regulator of G-protein signaling 2	up	1.17	0.02
OSM	oncostatin M	up	1.17	0.05
IFITM3	interferon induced transmembrane protein 3	up	1.16	0.00
MT1X	metallothionein 1X	up	1.16	0.02
RNF19B	ring finger protein 19B	up	1.16	0.00
ATOH8	atonal bHLH transcription factor 8	up	1.16	0.00
TREM1	triggering receptor expressed on myeloid cells 1	up	1.15	0.00
IRF1	interferon regulatory factor 1	up	1.15	0.02
BATF	basic leucine zipper ATF-like transcription factor	up	1.15	0.00
CDA	cytidine deaminase	up	1.15	0.00
TREML4	triggering receptor expressed on myeloid cells like 4	up	1.14	0.00
HIST1H2AC	histone cluster 1, H2ac	up	1.14	0.00
SCARF1	scavenger receptor class F member 1	up	1.14	0.00
PDE7B	phosphodiesterase 7B	up	1.13	0.01
AQP9	aquaporin 9	up	1.13	0.00
ELF1	E74 like ETS transcription factor 1	up	1.13	0.00
SP140	SP140 nuclear body protein	up	1.13	0.00
KREMEN1	kringle containing transmembrane protein 1	up	1.12	0.00
IL6R	interleukin 6 receptor	up	1.12	0.00
MGAM	maltase-glucoamylase	up	1.12	0.00
ADCY2	adenylate cyclase 2	up	1.12	0.00
TAP1	transporter 1, ATP binding cassette subfamily B member	up	1.12	0.01
IGDCC3	immunoglobulin superfamily DCC subclass member 3	up	1.12	0.01
SLC15A4	solute carrier family 15 member 4	up	1.12	0.00
KANK4	KN motif and ankyrin repeat domains 4	up	1.11	0.00
LRP10	LDL receptor related protein 10	up	1.11	0.00
S100A8	S100 calcium binding protein A8	up	1.11	0.02
GCH1	GTP cyclohydrolase 1	up	1.11	0.00
PIM3	Pim-3 proto-oncogene, serine/threonine kinase	up	1.11	0.00
DLC1	DLC1 Rho GTPase activating protein	up	1.10	0.02
PELI2	pellino E3 ubiquitin protein ligase family member 2	up	1.10	0.00
ATP6V1B1	ATPase H+ transporting V1 subunit B1	up	1.10	0.05
CRYAB	crystallin alpha B	up	1.10	0.00
CDC42	cell division cycle 42	up	1.10	0.00
PTP4A3	protein tyrosine phosphatase type IVA, member 3	up	1.09	0.00

IRS2	insulin receptor substrate 2	up	1.09	0.00
NOD2	nucleotide binding oligomerization domain containing 2	up	1.09	0.00
PLK3	polo like kinase 3	up	1.09	0.00
HOXA5	homeobox A5	up	1.09	0.00
ELAVL4	ELAV like neuron-specific RNA binding protein 4	up	1.09	0.01
KRT23	keratin 23	up	1.09	0.01
SMAP2	small ArfGAP2	up	1.09	0.00
ITPRIP	inositol 1,4,5-trisphosphate receptor interacting protein	up	1.09	0.00
COL8A1	collagen type VIII alpha 1 chain	up	1.09	0.00
KLHL34	kelch like family member 34	up	1.09	0.00
PLEKHO1	pleckstrin homology domain containing O1	up	1.09	0.01
RHOH	ras homolog family member H	up	1.08	0.00
GCLM	glutamate-cysteine ligase modifier subunit	up	1.08	0.00
CLDN14	claudin 14	up	1.08	0.00
FARSB	phenylalanyl-tRNA synthetase beta subunit	up	1.08	0.02
PAG1	phosphoprotein membrane anchor with glycosphingolipid microdomains 1	up	1.08	0.00
NKG7	natural killer cell granule protein 7	up	1.08	0.00
GBP4	guanylate binding protein 4	up	1.07	0.00
ST3GAL4	ST3 beta-galactoside alpha-2,3-sialyltransferase 4	up	1.07	0.00
SEZ6	seizure related 6 homolog	up	1.07	0.01
CACNG2	calcium voltage-gated channel auxiliary subunit gamma 2	up	1.07	0.01
STX11	syntaxin 11	up	1.07	0.00
OLIG1	oligodendrocyte transcription factor 1	up	1.07	0.00
NEDD9	neural precursor cell expressed, developmentally down-regulated 9	up	1.07	0.00
IPO11	importin 11	up	1.06	0.01
P2RY8	purinergic receptor P2Y8	up	1.06	0.00
ST8SIA4	ST8 alpha-N-acetyl-neuraminide alpha-2,8-sialyltransferase 4	up	1.06	0.00
BAZ1A	bromodomain adjacent to zinc finger domain 1A	up	1.06	0.00
INPP5A	inositol polyphosphate-5-phosphatase A	up	1.06	0.00
PRDM8	PR/SET domain 8	up	1.06	0.00
TNFSF13B	tumor necrosis factor superfamily member 13b	up	1.05	0.00
MSRB1	methionine sulfoxide reductase B1	up	1.05	0.01
E2F3	E2F transcription factor 3	up	1.05	0.00
HIST1H2BK	histone cluster 1, H2bk	up	1.05	0.00
CCL3	C-C motif chemokine ligand 3	up	1.04	0.01
KIF19	kinesin family member 19	up	1.04	0.03
PFKFB3	6-phosphofructo-2-kinase/fructose-2,6-biphosphatase 3	up	1.04	0.01
F5	coagulation factor V	up	1.04	0.00
SEC22B	SEC22 homolog B, vesicle trafficking protein (gene/pseudogene)	up	1.03	0.00
SMOX	spermine oxidase	up	1.03	0.01
NEDD4L	neural precursor cell expressed, developmentally down-regulated 4-like, E3 ubiquitin protein ligase	up	1.03	0.04
MLLT6	MLLT6, PHD finger domain containing	up	1.03	0.02
IRX2	iroquois homeobox 2	up	1.03	0.01
VAMP5	vesicle associated membrane protein 5	up	1.03	0.04

CARD17	caspase recruitment domain family member 17	up	1.02	0.00
GABARAPL2	GABA type A receptor associated protein like 2	up	1.02	0.00
PHC2	polyhomeotic homolog 2	up	1.02	0.00
TOM1	target of myb1 membrane trafficking protein	up	1.02	0.00
IL1RN	interleukin 1 receptor antagonist	up	1.02	0.03
BATF2	basic leucine zipper ATF-like transcription factor 2	up	1.02	0.00
TSC22D3	TSC22 domain family member 3	up	1.02	0.00
LIMS1	LIM zinc finger domain containing 1	up	1.02	0.01
NFE2L2	nuclear factor, erythroid 2 like 2	up	1.01	0.00
CASP4	caspase 4	up	1.01	0.00
CREBRF	CREB3 regulatory factor	up	1.01	0.00
CD48	CD48 molecule	up	1.01	0.01
DDIT4	DNA damage inducible transcript 4	up	1.01	0.00
KCNH2	potassium voltage-gated channel subfamily H member 2	up	1.00	0.00
LAMP3	lysosomal associated membrane protein 3	up	1.00	0.03
AGMAT	agmatinase	up	1.00	0.02
PTEN	phosphatase and tensin homolog	up	1.00	0.00
CARD19	caspase recruitment domain family member 19	up	1.00	0.00
ZDHHC18	zinc finger DHHC-type containing 18	up	1.00	0.00
TMEM140	transmembrane protein 140	up	1.00	0.00
IGFBP2	insulin like growth factor binding protein 2	down	-2.41	0.00
C8B	complement component 8, beta polypeptide	down	-2.15	0.00
ZNF589	zinc finger protein 589	down	-1.63	0.00
GPA33	glycoprotein A33	down	-1.59	0.01
TMEM74B	transmembrane protein 74B	down	-1.38	0.04
SHROOM3	shroom family member 3	down	-1.37	0.00
CA2	carbonic anhydrase 2	down	-1.31	0.01
ECHDC2	enoyl-CoA hydratase domain containing 2	down	-1.30	0.00
SLC47A1	solute carrier family 47 member 1	down	-1.29	0.01
RAPGEF3	Rap guanine nucleotide exchange factor 3	down	-1.27	0.01
MLPH	melanophilin	down	-1.25	0.01
GPD1	glycerol-3-phosphate dehydrogenase 1	down	-1.22	0.02
PNPLA7	patatin like phospholipase domain containing 7	down	-1.21	0.00
PROS1	protein S (alpha)	down	-1.21	0.00
HOXB7	homeobox B7	down	-1.20	0.00
COLEC12	collectin subfamily member 12	down	-1.19	0.01
SLC19A3	solute carrier family 19 member 3	down	-1.18	0.01
SH3PXD2A	SH3 and PX domains 2A	down	-1.18	0.00
ACKR3	atypical chemokine receptor 3	down	-1.18	0.01
TAGLN	transgelin	down	-1.17	0.03
SPIRE2	spire type actin nucleation factor 2	down	-1.17	0.00
GCHFR	GTP cyclohydrolase I feedback regulator	down	-1.15	0.01
ACACB	acetyl-CoA carboxylase beta	down	-1.14	0.01
LGALS3BP	galectin 3 binding protein	down	-1.14	0.01
FABP4	fatty acid binding protein 4	down	-1.14	0.02
FHL1	four and a half LIM domains 1	down	-1.13	0.01
ENPP3	ectonucleotide pyrophosphatase/phosphodiesterase 3	down	-1.13	0.02
RDH10	retinol dehydrogenase 10 (all-trans)	down	-1.12	0.03
PDE1B	phosphodiesterase 1B	down	-1.12	0.00

IDUA	iduronidase, alpha-L-	down	-1.12	0.00
GSTT1	glutathione S-transferase theta 1	down	-1.11	0.05
PLA2G16	phospholipase A2 group XVI	down	-1.11	0.01
TCEA3	transcription elongation factor A3	down	-1.10	0.01
GGA2	golgi associated, gamma adaptin ear containing, ARF binding protein 2	down	-1.10	0.00
TPM2	tropomyosin 2 (beta)	down	-1.09	0.05
COL9A2	collagen type IX alpha 2 chain	down	-1.09	0.00
MARCO	macrophage receptor with collagenous structure	down	-1.08	0.00
ABCC3	ATP binding cassette subfamily C member 3	down	-1.08	0.00
SLC4A11	solute carrier family 4 member 11	down	-1.06	0.01
RMDN3	regulator of microtubule dynamics 3	down	-1.06	0.00
PKD2L1	polycystin 2 like 1, transient receptor potential cation channel	down	-1.06	0.01
GLDN	gliomedin	down	-1.05	0.02
ST5	suppression of tumorigenicity 5	down	-1.05	0.00
EVL	Enah/Vasp-like	down	-1.05	0.00
MYB	MYB proto-oncogene, transcription factor	down	-1.05	0.01
LY6E	lymphocyte antigen 6 complex, locus E	down	-1.04	0.04
ICOS	inducible T-cell costimulator	down	-1.04	0.00
C15orf52	chromosome 15 open reading frame 52	down	-1.02	0.04
FOLR1	folate receptor 1	down	-1.02	0.01
PLA2G15	phospholipase A2 group XV	down	-1.02	0.01
DDIAS	DNA damage induced apoptosis suppressor	down	-1.02	0.01
PI4KAP2	phosphatidylinositol 4-kinase alpha pseudogene 2	down	-1.02	0.01
DGKQ	diacylglycerol kinase theta	down	-1.02	0.00
FAM156A	family with sequence similarity 156 member A	down	-1.02	0.01
SPARC	secreted protein acidic and cysteine rich	down	-1.01	0.01
SLC46A3	solute carrier family 46 member 3	down	-1.01	0.00
PON2	paraoxonase 2	down	-1.01	0.00
TRPV4	transient receptor potential cation channel subfamily V member 4	down	-1.00	0.00
FAM89A	family with sequence similarity 89 member A	down	-1.00	0.00
